# Delineating the Importance of Serum Opsonins and the Bacterial Capsule in Affecting the Uptake and Killing of *Burkholderia pseudomallei* by Murine Neutrophils and Macrophages

**DOI:** 10.1371/journal.pntd.0002988

**Published:** 2014-08-21

**Authors:** Minal Mulye, Michael P. Bechill, William Grose, Viviana P. Ferreira, Eric R. Lafontaine, R. Mark Wooten

**Affiliations:** 1 Department of Medical Microbiology and Immunology, University of Toledo College of Medicine, Toledo, Ohio, United States of America; 2 Department of Infectious Diseases, University of Georgia, Athens, Georgia, United States of America; Mahidol University, Thailand

## Abstract

Infection of susceptible hosts by the encapsulated Gram-negative bacterium *Burkholderia pseudomallei* (Bp) causes melioidosis, with septic patients attaining mortality rates ≥40%. Due to its high infectivity through inhalation and limited effective therapies, Bp is considered a potential bioweapon. Thus, there is great interest in identifying immune effectors that effectively kill Bp. Our goal is to compare the relative abilities of murine macrophages and neutrophils to clear Bp, as well as determine the importance of serum opsonins and bacterial capsule. Our findings indicate that murine macrophages and neutrophils are inherently unable to clear either unopsonized Bp or the relatively-avirulent acapsular bacterium *B. thailandensis* (Bt). Opsonization of Bp and Bt with complement or pathogen-specific antibodies increases macrophage-uptake, but does not promote clearance, although antibody-binding enhances complement deposition. In contrast, complement opsonization of Bp and Bt causes enhanced uptake and killing by neutrophils, which is linked with rapid ROS induction against bacteria exhibiting a threshold level of complement deposition. Addition of bacteria-specific antibodies enhances complement deposition, but antibody-binding alone cannot elicit neutrophil clearance. Bp capsule provides some resistance to complement deposition, but is not anti-phagocytic or protective against reactive oxygen species (ROS)-killing. Macrophages were observed to efficiently clear Bp only after pre-activation with IFNγ, which is independent of serum- and/or antibody-opsonization. These studies indicate that antibody-enhanced complement activation is sufficient for neutrophil-clearance of Bp, whereas macrophages are ineffective at clearing serum-opsonized Bp unless pre-activated with IFNγ. This suggests that effective immune therapies would need to elicit both antibodies and Th1-adaptive responses for successful prevention/eradication of melioidosis.

## Author Summary

*Burkholderia pseudomallei* is the causative agent of melioidosis, which is a major cause of septic death in endemic areas of Southeast Asia and northern Australia. This range now appears to be expanding, and with the increased incidence of diabetes, which is a major predisposing factor for *B. pseudomallei* infections, it is believed these cases will continue to increase. This organism is also categorized as a Tier 1 select agent with great potential for misuse as a bioweapon. Thus, there is great interest in developing vaccines and other immunotherapies for melioidosis. To better design these treatments, it is essential to understand which immune cells are capable of controlling *B. pseudomallei*, particularly during early stages of infection, and which immune mediators are required for these cells to optimally eradicate these bacteria. In this work, we identified that both macrophages and neutrophils were capable of internalizing and killing *B. pseudomallei*, but required different immune mediators to allow these effects. Complement and antibody-mediated opsonization was sufficient to promote uptake and killing of *B. pseudomallei* by neutrophils, whereas these opsonins could only enhance uptake by macrophages; macrophage-mediated killing required IFNγ. These findings suggest that successful therapies must elicit complement-activating antibodies and IFNγ to eradicate/prevent melioidosis.

## Introduction

The causative agent of melioidosis, *Burkholderia pseudomallei* (Bp), is an encapsulated, motile, Gram-negative bacillus found free-living in soil [1]. Melioidosis is endemic to tropical and subtropical areas, and is most often described in southeast Asia and northern Australia [2], [3]. It is a major cause of septic deaths in these regions, with mortality rates of 50% in Thailand [4] and 20% in Australia [5], even after antibiotic treatment. Recently, increasing occurrences of melioidosis have been observed in other parts of the world, including west and east Africa, the Caribbean, Central and South America and the Middle East [6]–[8]. Melioidosis has a wide spectrum of clinical manifestations ranging from a simple inapparent infection to a fatal septicemia, with the largest influences being the route of infection, size of inoculum, and susceptibility of the host. Diabetes is considered the most significant risk factor, with studies suggesting that between 23–60% of melioidosis patients are diabetic and that this condition may increase the relative risk of infection by ≥20-fold [9], [10]. The most severe and often rapidly fatal form of the disease is acute septicemia or pulmonary melioidosis, which is characterized by abscess formation commonly in the lungs, liver, and/or spleen, along with bacteremia. In many cases, even vigorous antibiotic and supportive treatments do not prevent mortality [3], [11]. Chronic melioidosis is characterized by a persistent infection that can recrudesce at varying times after the initial infection, with the longest being 62 years post-infection [12]. Skin inoculation is thought to be the primary cause of natural Bp infection in endemic areas following exposure to muddy soils or surface water, such as rice paddy fields [13], [14]. Though, the inoculum required to cause disease through this route is relatively large [6], inhalation of only 5–100 organisms can lead to severe disease in mice, with mortality observed within 5–6 days post-infection [15]–[17]. Owing to the low inoculum required to cause high mortality via inhalation, lack of a preventive vaccine and the unreliable nature of the existing antibiotic treatments, Bp is categorized as a Tier 1 select agent by the Center for Disease Control and Prevention (CDC). Thus, there is a worldwide interest in better understanding the interaction of Bp with the host immune system and developing protective therapies for melioidosis.

Although Bp can thrive as a free-living saprophyte in moist environments, it can also successfully persist within both phagocytic and non-phagocytic cells, including neutrophils, macrophages, dendritic cells, epithelial cells and endothelial cells [18]–[20]. Bp is reported to escape the endosome/phagosome of host cells within ≥15 min of cell entry and persist/replicate within the cytoplasm of those cells [21]. Bp can subsequently spread cell-to-cell by polymerizing host actin that it “rides” to protrude through neighboring cell membranes, and can also cause giant-cell formation, both of which allow bacterial spread with minimal exposure to the extracellular environment [22]. Bp utilize a variety of virulence factors, the best-studied of which includes type III and type VI secretion systems, to enter, survive, and persist within host cells [23]–[25]. While these infections do elicit B and T cell responses, the importance of the adaptive immune response in clearing infection is somewhat unclear. The presence of high antibody titers is not protective in certain cases, suggesting that humoral immunity can be insufficient [26], [27]. Also, HIV patients do not display enhanced susceptibility to melioidosis, even though they possess severely impaired T cell responses [28]. It is speculated that the innate cellular responses are critical for controlling the development of melioidosis, especially considering the rapid progression of Bp infections. Many of the existing co-morbidities for Bp infection (e.g. diabetes) are associated with defects in the innate immune responses, further suggesting a crucial role for these mechanisms [5], [29]–[38]. However, the relative importance of many of these innate components is largely unknown.

The importance of macrophages in Bp pathogenicity is somewhat unclear. Depletion of macrophages from BALB/c or C57BL/6 mice significantly increases mortality during experimentally induced melioidosis compared with untreated mice [39], [40]. Bp-infected C57BL/6 mice (i.e. melioidosis-resistant) are observed to recruit macrophages to infection sites more efficiently than BALB/c mice (i.e. melioidosis-susceptible), and it is speculated that C57BL/6 macrophages are more effective at controlling Bp infection compared to BALB/c macrophages, leading to the subsequent development of chronic (C57BL/6) versus acute (BALB/c) disease manifestations [41]. Though these data suggest macrophages play an important protective role in early defense against infection, Bp also possess a number of mechanisms to evade macrophage-mediated clearance [42]. Bp can suppress the production of IFNγ and IFNβ by infected macrophages, which subsequently leads to inadequate iNOS expression [43]–[45]. The inability to generate sufficient nitric oxide, which is a major macrophage-elicited killing mechanism for intracellular bacteria, is believed to contribute to Bp persistence within macrophages. Additionally, components of the Bp type III secretion system have been shown to reduce autophagy in infected cells, which is an important mechanism for clearing intracellular bacteria after escaping from phagosomal compartments [46], [47]. Thus, the relative importance of macrophages in clearing Bp infections versus potentially serving as a reservoir of persistent infection is currently unclear.

In contrast, a number of *in vivo* findings suggest that neutrophils are important for controlling melioidosis. Depletion of neutrophils from mice resulted in an acute and more rapidly lethal Bp infection [48], indicating the importance of neutrophils in early clearance. Neutrophils are recruited to and interact with Bp within the lung tissue of infected mice [48], [49], and mice lacking NADPH oxidase, an important enzyme in the generation of the neutrophil respiratory burst, were more susceptible to Bp infection [39]. More indirectly, the reported co-morbidities for melioidosis also suggest the importance of neutrophils during Bp infection. Diabetes mellitus, the primary pre-disposing condition for melioidosis, is associated with impaired chemotaxis, phagocytosis, oxidative burst, and killing activity of neutrophils [29]–[37]. Similar defects in neutrophil function have also been observed with other co-morbidities, such as increased alcohol consumption, renal failure and thalassemia [6], [50]–[52]. Increased incidence of melioidosis is also noted in neutropenic patients [53], [54], as well as patients with chronic granulomatous disease [55], [56]. Interestingly, the neutrophil-differentiating cytokine granulocyte colony-stimulating factor (G-CSF) has been used as a treatment for melioidosis, with mixed results; it is reported to reduce mortality of melioidosis patients in Australia [57], but is only associated with prolonged survival in Thailand patients [58]. Thus, all these *in vivo* reports suggest the importance of neutrophils in controlling melioidosis, but the mechanisms of how neutrophils affect Bp infection are not clear.

A number of studies have been performed *in vitro* to detect the effectiveness of macrophages and neutrophils in clearing Bp, using various culture conditions and producing mixed results [18], [35], [59]–[61]. A recent study published from our lab showed that human neutrophils can efficiently kill Bp once it is opsonized with human serum [62]. However there are no reports comparing the relative importance of neutrophils and macrophages in clearance of Bp, including the effects of different soluble immune opsonins in promoting bacterial uptake and killing. Thus, the overall goal of our study is to compare the relative abilities of macrophages and neutrophils to clear Bp and to determine whether certain immune opsonins are needed to mediate clearance. We also assessed the relative importance of bacterial capsule by including studies using a Bp capsule mutant [63], [64], as well as the closely-related but relatively-avirulent acapsular *Burkholderia thailandensis* (Bt) species, thus allowing for delineation of capsular versus other genetic effects on resisting phagocyte clearance. The findings of these studies will contribute to the understanding of the immune mediators required for cellular clearance of Bp, as well as provide a roadmap of the type(s) of immunity that need to be elicited by Bp-directed preventative therapies.

## Materials and Methods

### Bacterial strains and culture

All of the bacterial strains used in this study are listed in Table 1, and are also briefly described as follows. *Burkholderia pseudomallei* (Bp) 1026b [65], Bp strain DD503 [66], and *Burkholderia thailandensis* (Bt) E264 [67], [68] were generously provided by Dr. Donald Woods (University of Calgary). Bp 1026b is a highly virulent clinical isolate from a Thai patient with septicemic melioidosis [65] and Bt E264 is a relatively avirulent environmental isolate from a rice field soil sample in central Thailand [67], [68]. Bp DD503 is a mutant of 1026b that lacks the multidrug efflux system, making this strain more susceptible to aminoglycoside and macrolide antibiotics, and thus more amenable to genetic manipulation and subsequent antibiotic selection [66]; DD503 does exhibit similar virulence in hamsters as the 1026b parent strain [63]. The SZ210 acapsular mutant [63] and the BP2683 LPS non-expressing mutant (ΔLPS) [69] was engineered by allelic exchange with DD503, and were generously provided by Paul Brett and Mary Burtnick (University of South Alabama). The serum-sensitive *Escherichia coli* strain K12 (substrain W3110; ATCC 27325) was used in certain experiments as a positive control for complement- or phagocyte-mediated killing. All bacterial strains were cultured on tryptic soy agar (TSA) plates aerobically at 37°C for 16–18 h prior to use. Bacterial suspensions were prepared in PBS by scraping the bacteria from TSA plates. Bacterial stocks were enumerated using a spectrophotometer at OD_600_ and the bacterial numbers were confirmed by dilution plating. All studies involving Bp were performed in the CDC-certified BSL3 laboratory at the University of Toledo Health Sciences Campus.

**Table 1:** Bacterial strains.

Bacterial Strains	Characteristics	Source or reference
*B. pseudomallei* 1026b	Clinical isolate; Thailand	[65]
*B. pseudomallei* DD503	1026b derivative; Δ(*amrR*-*amrAB*-*oprA*): Km^s^, Pm^r^	[66]
*B. pseudomallei* SZ210	DD503 derivitive; Δ*wcbB*; : Km^s^, Pm^r^; lacks capsule	[63]
*B. pseudomallei* BP2683	DD503 derivitive; Δ*rmlD*: Km^s^, Pm^r^; lacks LPS	[69]
*B. thailandensis* E264	Environmental isolate; Thailand	ATCC 700388 [67], [68]
*E. coli* K12	Substrain W3110	ATCC 27325

### Mice

Female C57BL/6 and BALB/c mice were obtained from the National Cancer Institute (Bethesda, MD). Mice were housed in the Department of Laboratory Animal Research at the University of Toledo Health Sciences Campus, and all procedures were in accordance with and approved by the University of Toledo Institutional Animal Care and Use Committee (IACUC).

### Ethics statement

Animal experiments were performed in strict accordance with the recommendations in the Guide for the Care and Use of Laboratory Animals of the National Institutes of Health. The protocol was approved by the University of Toledo Institutional Animal Care and Use Committee (IACUC; protocol #104757).

### Antibiotic sensitivity assays

To determine the relative antibiotic sensitivities of the different *Burkholderia* strains over time, 10^5^ bacteria were incubated in macrophage co-culture media (RPMI 1640 with 10% FBS; i.e. complete RPMI) containing different concentrations of kanamycin for a total of 24 h. Bacterial viability was assessed at the indicated times by serial dilution and plating onto TSA plates. Bacterial numbers were enumerated as colony forming units (CFU) per ml.

### Isolation of primary murine macrophages

Bone marrow from the femurs and tibias of C57BL/6 (B6) or BALB/c mice was cultured as previously described [70]. Briefly, bone marrow cells were cultured in complete RPMI supplemented with L929-conditioned medium for 7 days at 37°C. Expanded macrophages were recovered by scraping in ice-cold PBS, enumerated by direct counting using a hemacytometer, and seeded into 24-well tissue culture plates at a density of 2×10^5^ cells/well in complete RPMI. C57BL/6-derived macrophages were used for all studies unless otherwise indicated.

### Isolation of primary murine neutrophils

Primary neutrophils were isolated from murine bone-marrow as previously reported [71]. Briefly, bone marrow from femur and tibia bones was harvested in HBSS and red blood cells (RBCs) were lysed using RBC-lysis buffer. The leukocyte suspension was centrifuged at 500× g for 10 min, resuspended in HBSS and then overlayed on a Percoll (Sigma-Aldrich P4937) gradient containing 55%, 65% and 75% layers. The density gradient centrifugation was performed at 500× g for 30 min with brakes off and the cells at the 65/75 interface were harvested as neutrophils. Isolated neutrophils were washed and enumerated using haemocytometer. Their purity was determined using a differential hematology analyzer (VetScan HM5, Abaxis Veterinary Diagnostics), Wright–Giemsa differential staining, and flow cytometry using anti-mouse Ly6G antibodies [72]. Based on these values, the neutrophils were resuspended to obtain a final density of 4×10^5^ cells/ml. After allowing the cells to adhere to the particular assay plate and eluting non-adherent cells, the remaining cell population contained ≥90% pure neutrophils. C57BL/6-derived neutrophils were used for all studies unless otherwise mentioned.

### Bacterial opsonization

Guinea pig serum used as a complement source was purchased from MP Biomedicals (Cat# 642831). Guinea pig serum shows enhanced complement-dependent bactericidal activity compared to mouse serum [73], which possesses classical pathway components that are notoriously unstable compared to other mammalian species, and hence is difficult to work with [74]. Guinea pig complement components are recognized by and promote expected responses in mouse macrophages [75].

Before opsonization, bacteria from overnight cultures on TSA plates were suspended in PBS and enumerated by spectrophotometry. These suspended bacteria were incubated with different concentrations of guinea pig serum in PBS containing 1.2 mM CaCl_2_ and 4.25 mM MgCl_2_ (PBS^++^) at 37°C for 30 min. Guinea pig serum inactivated by heating at 56°C for 30 min (heat inactivated; HI) was used as a negative control. For antibody-mediated opsonization, bacteria were incubated with heat-inactivated mouse antiserum for 10 min at 37°C before adding guinea pig serum (i.e. complement source) for 30 min. The antisera were generated by immunizing groups of mice i.p. with 10^7^ heat-killed Bp or Bt in complete Freund's adjuvant, followed by two similar boosting immunizations in incomplete Freund's adjuvant at 3 week intervals. Bacteria-specific antibodies were initially confirmed by Western blot and ELISA using sonicated *Burkholderia* sp., and antibody levels were quantified by comparison to standards produced using known amounts of purified Ig of the appropriate isotype to wells coated with rabbit anti-mouse IgG+IgM+IgA antibodies per our previously published protocol [76]; these antisera contained Burkholderia-specific IgG levels at ≥2 mg/ml, including high levels of LPS-specific (Bp and Bt) and capsule-specific (Bp only) IgG. The specificity of the antisera to bind intact bacteria was determined against the respective bacterium by flow cytometry analyses (data not shown).

### Complement deposition analyses

Bacteria were opsonized with serum as described above. Opsonized bacteria were washed and then stained using 1∶500 Fluorescein isothiocynate (FITC)-conjugated polyclonal anti-guinea pig C3 antibody (Cat # 55385; Cappel), then washed and fixed with 2% paraformaldehyde. Labeled bacteria were analyzed by flow cytometry using a BD FACSCalibur and results reported as mean fluorescence intensity (MFI).

### Direct killing of *Burkholderia* strains

Bacteria (10^8^) were incubated with different concentrations of guinea pig serum in PBS^++^ at 37°C. At the indicated times, samples were serially diluted and plated onto TSA plates to determine the colony forming units (CFU)/ml.

### Bacterial uptake and intracellular growth

#### Macrophages

Bone marrow-derived macrophages were seeded in 24-well plates in complete RPMI. Cells were allowed to attach overnight at 37°C in the presence of 5% CO_2_ before infecting with unopsonized or opsonized bacteria at a multiplicity of infection (MOI) = 1 in duplicates. Plates were centrifuged at 300× g for 5 min to synchronize infection and incubated at 37°C in 5% CO_2_ for 1 h. Monolayers were washed 3 times with media to remove extracellular bacteria before replenishing with complete RPMI containing 500 µg/ml of kanamycin to kill extracellular bacteria. After 2 h of incubation in kanamycin-containing medium, the cells were washed thoroughly to remove the residual antibiotic. At this time (3 h post-infection), the macrophages were either lysed with 0.5% saponin in PBS and dilution plated on TSA to determine CFU/ml (i.e. bacterial uptake) or fresh media was added and infected cells were incubated until 10 h post-infection (to determine bacterial survival). Bacterial uptake is reported as the internalization index, which is the percentage of bacteria recovered at 3 h compared to the bacterial input (0 h). Bacterial survival is represented as the survival index, which is the fold-change in bacterial numbers at 10 h compared to 3 h (internalization).

To determine if macrophages pre-stimulated with IFNγ showed enhanced killing of opsonized compared to unopsonized bacteria, macrophages were pre-activated with 100 U of murine IFNγ (R&D Systems) for 16–18 h prior to infection. Intracellular growth assays were then performed as described above, and internalization and survival indices were determined.

#### Neutrophils

Murine neutrophils were seeded to obtain 4×10^5^ cells per well in 48-well plates containing complete RPMI with 10% FBS and incubated at 37°C in 5% CO_2_ for 20 min. Non-adherent cells were removed by washing before infecting with the bacteria at MOI = 1. Intracellular growth assays with neutrophils were performed under two conditions: 1) No blockage of reactive oxygen species (ROS) production when treated with dimethylsulfoxide (DMSO; vehicle control), and 2) blockage of ROS production when treated with diphenyleneiodonium (DPI), which is a potent inhibitor of NADPH oxidase [77]. Blockage of ROS allowed for the most accurate assessment of bacterial uptake by excluding any rapid bacterial killing by neutrophils, which could mask the true bacterial internalizations values.

Neutrophils (+/−10 µM DPI or DMSO) were infected with bacteria at MOI = 1. Plates were centrifuged at 300× *g* for 5 min to synchronize infection and incubated at 37°C in 5% CO_2_ for 10 min before washing 3 times with media to remove extracellular bacteria. At 10 min post-infection, neutrophils were lysed using 0.5% saponin solution, and internalized bacteria were enumerated by dilution plating to determine bacterial uptake. Parallel co-cultures of neutrophils were lysed at 2 h post-infection and bacteria again dilution plated to determine bacterial killing. Bacterial uptake was reported as the internalization index, which is the percentage of bacteria recovered at 10 min compared to the bacterial input (0 h). Bacterial survival was represented as survival index which is calculated as the fold-change in bacterial numbers at 2 h compared to those internalized at 10 min post-infection. The 10 min and 2 h time points were chosen to determine internalization and survival in neutrophils, respectively, due to faster activation kinetics and short life span of neutrophils compared to macrophages [78].

### Quantification of neutrophil respiratory burst

Luminol-based chemiluminescence assays were performed to assess the reactive oxygen species (ROS) production by neutrophils. Isolated murine neutrophils were cultured in complete RPMI at 2×10^6^ cells per well in a 6-well plate for 2 h. Neutrophils were then re-seeded at 2×10^5^ cells per well in a 96-well plate in PBS^++^ in the presence or absence of DPI (10 µM). Neutrophils were pretreated with luminol (200 µM 3-aminophthalhydrazide in 0.1 M NaOH), horseradish peroxidase (50 U/ml in sterile water) and cytochalasin B (5 ug/ml) at 37°C in 5% CO_2_ for 20 min before infecting with differentially opsonized bacteria at MOI = 5. Cells were immediately analyzed on a FLUOstar Omega plate reader (BMG Labtech) with luminescence detection every 20 s for 10 min. The time required to start detection of chemiluminescence post-infection (40 s) was considered as the starting point in the data figures.

### Statistical analyses

Graphpad Instat (La Jolla, CA) was used for statistical analyses of all datasets using one-way ANOVA with the Tukey's post-hoc test. *P*-values of ≤0.05 were considered statistically significant.

## Results

### Bp and Bt possess different kanamycin sensitivities

**Figure 1:**
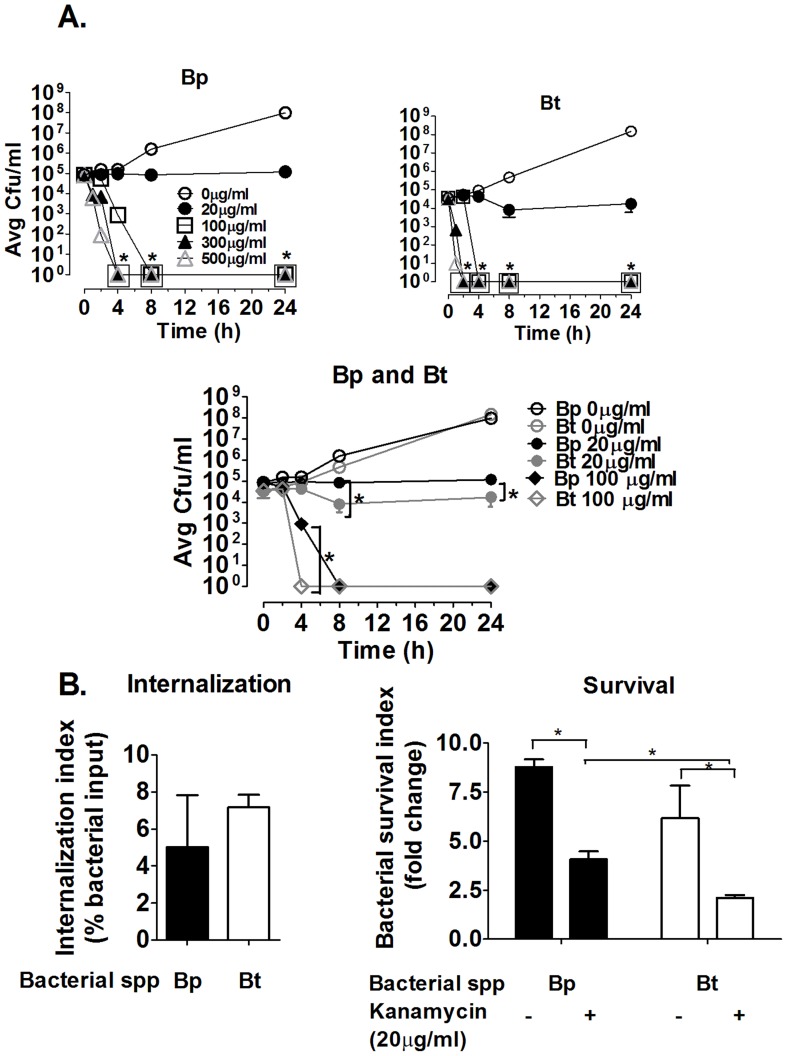
Effects of kanamycin on extracellular and intracellular killing of Bp and Bt. 10^5^ Bp (**A**) or Bt (**B**) were incubated in complete RPMI containing the indicated concentrations of kanamycin for a total of 24 h. Bacterial viability was assessed at the indicated times by serial dilution and plating onto TSA plates. Bacterial numbers were enumerated as colony forming units (CFU)/ml. For **A**–**B**, * indicated comparison to respective 0 µg/ml. **C**) Differences between Bp and Bt at 20 and 100 µg/ml kanamycin. * p<0.05 as determined by one-way ANOVA, error bars represent standard error mean; n = 4. **D–E)** C57BL/6 bone marrow-derived macrophages were infected with bacteria (MOI = 1) for 1 h before killing extracellular bacteria using 500 µg/ml kanamycin. Some co-cultures were harvested at 3 h post-infection to determine internalization (**D**). For the remaining cells, half were replenished with fresh media and the other half with medium containing 20 µg/ml of kanamycin at 3 h. On harvesting these cells at 10 h post-infection for enumeration, survival indices were determined (**E**) (n≥6). * p<0.05 as determined by one-way ANOVA, error bars represent standard error mean.

Because intracellular growth assays were used in the majority of these experiments, it was important to determine the optimum antibiotic concentration required for killing extracellular Bp and Bt in the minimal amount of time. Both Bp and Bt required relatively high kanamycin concentrations for efficient killing within 1–2 h incubation (Figure 1A–B), although Bt was more susceptible to kanamycin compared to Bp, as indicated by Bt killing at earlier time points compared to Bp (Figure 1C). These studies indicated that both bacteria required incubation for at least 1–2 h in 500 µg/ml of kanamycin to get complete killing, and these conditions were used for performing intracellular growth assays.

### Presence of even low levels of antibiotics in culture media significantly alters intracellular killing by macrophages

Intracellular growth assays were performed in the presence/absence of relatively low residual kanamycin concentrations to determine whether it affects intracellular growth assays. In the presence of 20 µg/ml, which is >25× less than the levels needed to efficiently kill either strain in media and elicits little to no killing of Bp or Bt (Figure 1C), no effects were observed in the intracellular uptake by macrophages compared to non-residual antibiotic controls (Figure 1D). Importantly, the residual kanamycin did reduce the intracellular levels of both bacteria over time, and there were significant effects on intracellular clearance between Bp and Bt, with Bt showing reduced survival compared to Bp, which was not evident in the absence of antibiotics in the macrophage co-culture media (Figure 1E). Cultures to determine if extracellular bacteria remained after the initial antibiotic treatments and before lysis of macrophage monolayers indicated that very few bacteria were present in the supernatants, and that these extracellular bacteria appear ≥8 h post-infection and coincide with the time that a subset of Bp were noted to emerge from macrophages via actin-mediated motility (data not shown). These findings suggest that the use of even relatively low antibiotic levels for more than a few hours can mediate a “pseudo” clearance of both bacteria by macrophages, with increased survival of Bp being most likely due to their enhanced resistance to kanamycin compared to Bt (Figure 1C) [79]. Several studies have reported similar effects, where residual antibiotics are taken up by and concentrated within macrophages [80]–[83]. Based on these findings, all subsequent assays only use a high dose of kanamycin for 2 h to kill extracellular bacteria before washing to remove all antibiotics for the remainder of the intracellular assessment.

### Bp and Bt show similar levels of uptake and persistence in murine macrophages *in vitro*

**Figure 2:**
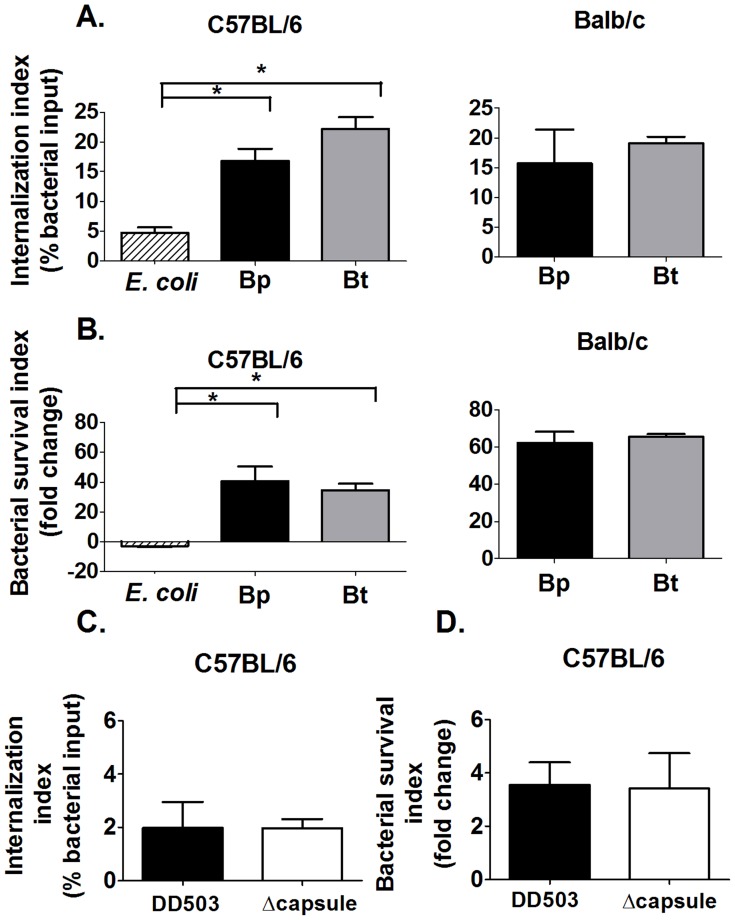
Interaction of non-opsonized *Burkholderia* strains with macrophages. Macrophages derived from the bone marrow of C57BL/6 and Balb/c mice were infected with the indicated bacteria (MOI = 1) for 1 h before killing extracellular bacteria using kanamycin. Macrophages were harvested at 3 and 10 h post-infection to determine bacterial internalization (**A** and **C**) and intracellular survival (**B** and **D**), respectively; bacterial numbers were determined by dilution plating (n≥6). * p<0.05 as determined by one-way ANOVA, error bars represent standard error mean.

Macrophages play an important role in clearance of many pathogenic bacteria, however, Bp has been shown to evade macrophage-mediated clearance and persist within these cells [84]. The inherent abilities of virulent Bp and relatively avirulent Bt to internalize and persist within macrophages derived from melioidosis-resistant (C57BL/6) and melioidosis-susceptible (BALB/c) mouse lines were assessed using intracellular growth assays [85]. Macrophages were capable of internalizing and clearing the non-virulent *E. coli* strain K12 by 10 h post-infection, indicating that the macrophages were functional (Figure 2A–B). In the absence of opsonins, Bp and Bt were internalized at similar rates by both types of macrophages (Figure 2A). At later times, Bp and Bt showed a similar significant increase in bacterial numbers in both C57BL/6 and BALB/c-derived macrophages (Figure 2B). Intracellular growth assays were also performed in two murine macrophage-like cell lines, J774 and RAW264.7, with results similar to those displayed by the primary macrophages (data not shown). These studies indicated that there are no differences in the abilities of Bp and Bt to be internalized or persist within different macrophage types, and that infection of murine macrophages *in vitro* does not reflect the differences in virulence observed between Bp and Bt *in vivo*.

### Bacterial capsule does not confer resistance to phagocytosis and survival of Bp within murine macrophages *in vitro*

Intracellular growth assays were also performed using the Bp DD503 parental strain and a congenic acapsular mutant in the absence of serum opsonins. Both Bp and the acapsular mutant showed similar levels of internalization, although lower than wild type strains that possess the efflux pump, as well as survival within murine macrophages (Figure 2C–D). The finding that Bp and the acapsular mutant (both are on the DD503 genetic background) are internalized at similar levels suggests that Bp capsule does not interfere with bacterial internalization by macrophages. Capsule also does not appear to directly affect Bp survival in macrophages, as indicated by similar survival indices between Bp and the acapsular mutant.

### Bp and Bt show similar inherent levels of uptake and persistence by murine neutrophils *in vitro*

**Figure 3:**
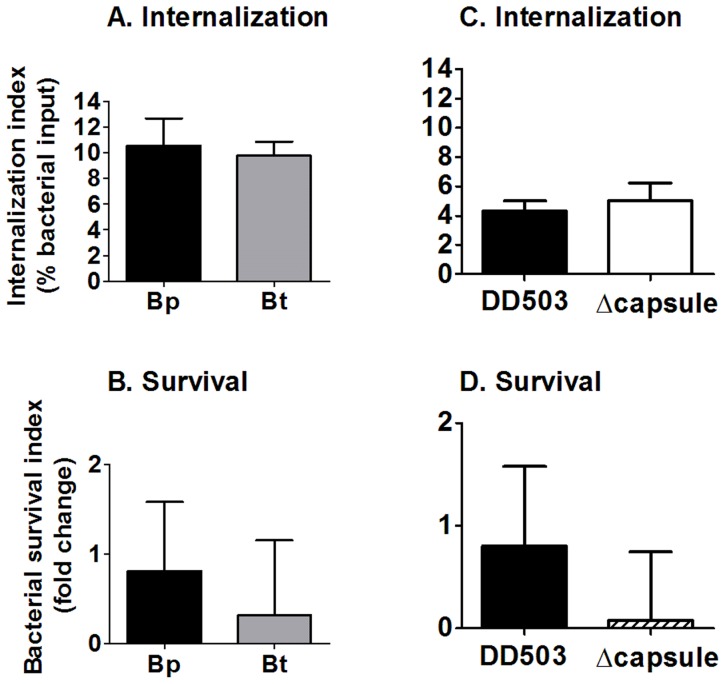
Interaction of non-opsonized *Burkholderia* strains with neutrophils. C57BL/6 bone marrow-derived neutrophils were infected with the indicated bacteria (MOI = 1) and the extracellular bacteria were removed by washing 10 min post-infection. Infected neutrophils were harvested at (**A** and **C**) 10 min to measure bacterial uptake and at (**B** and **D**) 2 h to measure bacterial survival. Bacterial numbers were determined by dilution plating (n≥6). * p<0.05 as determined by one-way ANOVA; error bars represent standard error mean.

Murine primary neutrophils were also co-cultured with Bp and Bt in the absence of serum opsonins to determine whether they displayed inherent differences in uptake and clearance by neutrophils. No differences were observed in the uptake of Bp and Bt at 10 min post-infection, or in the survival indices of Bp and Bt within neutrophils at 2 h post-infection (Figure 3A–B). These findings indicate that Bp and Bt possess a similar inherent resistance to neutrophil-mediated killing.

### Bacterial capsule does not confer resistance to phagocytosis and survival of Bp within murine neutrophils *in vitro*

Intracellular growth assays were also performed with Bp and an acapsular mutant to delineate whether capsule provided any inherent evasion properties against neutrophils. Both Bp and the acapsular mutant showed similar levels of bacterial internalization and intracellular survival, which is similar to the pattern displayed by Bp and Bt (Figure 3C–D). These findings indicate that the capsule produced by Bp does not directly affect phagocytosis and intracellular killing by neutrophils.

### Bp displays significantly reduced complement deposition compared to Bt or an acapsular mutant

**Figure 4:**
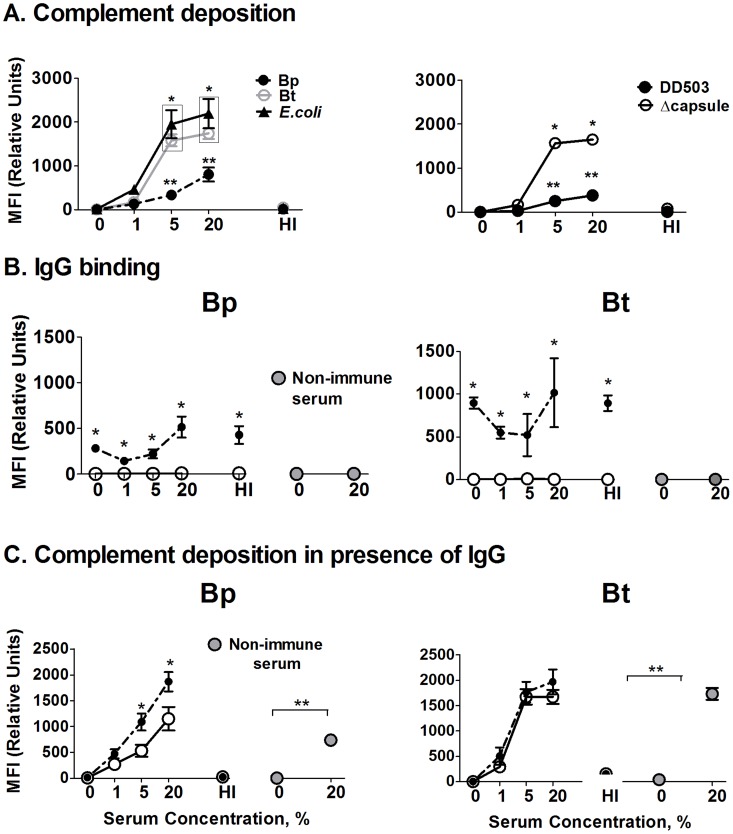
Assessment of complement deposition and antibody binding on *Burkholderia* strains. **A**) 10^7^ bacteria were incubated with the indicated serum concentrations for 30 min at 37°C, washed, and stained with FITC-conjugated polyclonal anti-C3 antibody. Triplicate samples were then fixed and C3 levels quantified by flow cytometry (n = 3). (* p<0.05 compared to respective Bp values, ** p<0.05 compared to Bp 0% as determined by one-way ANOVA; error bars represent standard error mean); (HI – Heat inactivated serum). **B**) 10^7^ bacteria were incubated with a complement source alone (white circles) or together with antiserum (black circles; 1∶200) generated against heat-killed Bp or Bt with the indicated amount of complement (% final concentration) for 30 min, and then washed before labeling with FITC-anti-mouse IgG. Triplicate samples were fixed and IgG levels quantified by flow cytometry (n = 3). * p<0.05 compared to respective no antiserum values as determined by one-way ANOVA; error bars represent standard error mean. **C**) Bacteria were incubated with antisera for 10 min as in **B**, followed by incubation with the indicated serum concentrations for 30 min before washing and staining for C3 detection as in **A**. Triplicate samples were fixed and C3 levels quantified by flow cytometry (n = 3). * p<0.05 compared to respective no 0% normal serum values as determined by one-way ANOVA; error bars represent standard error mean.

Phagocyte recognition of pathogens *in vivo* is assisted by various soluble immune mediators which aid in the recognition, ingestion, and killing of bacterial pathogens (i.e. opsonins). Two of the best-known opsonins include complement system components and certain antibodies isotypes that can assist both macrophages and neutrophils in phagocytosis. To begin addressing the importance of these opsonins, initial studies were performed to quantify differences in complement deposition and antibody binding between the different bacterial strains *in vitro*. Flow cytometric analyses indicated that acapsular Bt incubated in normal serum displayed similar levels of complement component C3 deposition as the positive control (*E. coli*) at all serum concentrations, whereas Bp demonstrated significantly reduced C3 deposition at 5% and 20% serum concentrations compared to Bt and *E. coli* (Figure 4A, left panel). No C3 deposition was observed on any bacteria opsonized with heat-inactivated (HI) serum (negative control). These data indicate that Bp is inherently more resistant to complement deposition than Bt. To more directly delineate whether the Bp capsule may mediate these differences in C3 deposition, similar flow cytometry studies were performed using the Bp acapsular mutant. The parent Bp DD503 displayed similar low C3 binding as wild type Bp 1026b, whereas the acapsular mutant showed significantly greater C3 binding than the parental DD503, with levels similar to that of Bt (Figure 4A, right panel). In agreement with previous reports [62], [63], these findings indicate that Bp is more resistant to C3 deposition than Bt, which appears largely due to the protective ability of their polysaccharide capsule.

### Antibody binding enhances complement deposition on Bp

Antibodies represent another important opsonin which can both directly promote phagocytosis/killing, as well as significantly enhance complement activation/deposition on certain bacterial pathogens. To directly evaluate antibody binding to the different strains, the indicated bacteria were incubated in Bp- or Bt-generated antisera in the presence or absence of a complement source, and IgG levels on bacterial surface detected using flow cytometry. Both Bp and Bt showed significant surface binding of IgG present in the respective antisera, whereas no antibody binding was observed in the presence of non-immune serum (Figure 4B). When a complement source was added together with the antisera, no differences were seen other than some increased binding at the higher complement levels, indicating there were no detrimental effects on antibody binding. We then assessed whether antibodies binding can enhance complement binding on these bacterial surfaces (Figure 4C). Bp-specific antibodies did enhance complement deposition on Bp in the presence of >5% normal serum, whereas Bt-specific antibodies did not confer any additional complement deposition over normal serum alone. These findings suggest that antibody is important for enhancing complement deposition on encapsulated Bp, as opposed to the non-encapsulated Bt, and is capable of increasing these complexes to levels similar to that of non-encapsulated Bt strain (Figure 4C).

### All three *Burkholderia* strains are equally resistant to complement-mediated direct killing

**Figure 5:**
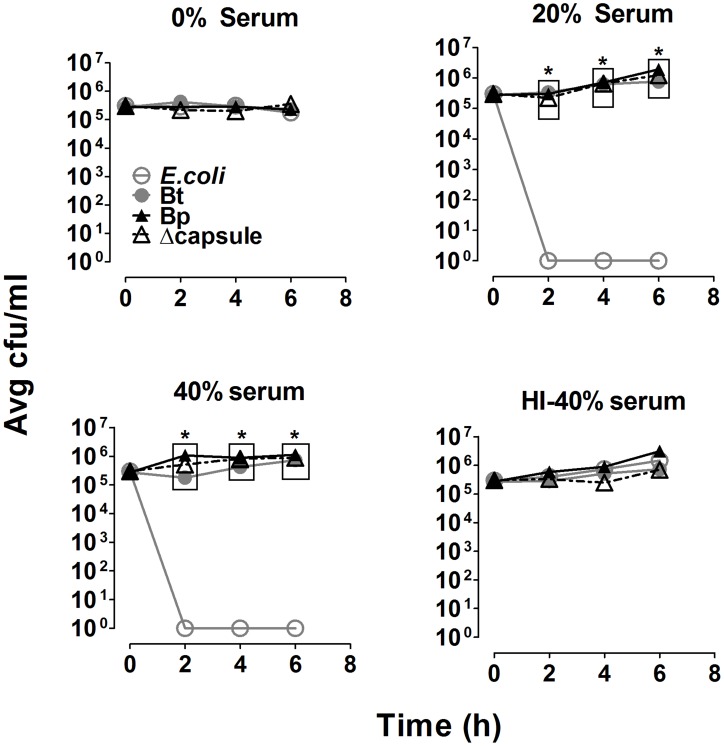
Complement-mediated direct bacterial killing. 10^8^ bacteria were incubated with the indicated serum concentrations in PBS^++^ at 37°C. At the indicated times, samples were recovered and bacteria were enumerated by dilution plating (n = 4). * p<0.05 compared to respective *E. coli* values, as determined by one-way ANOVA; error bars represent standard error mean.

To assess the effects of complement and bacterial capsule on direct killing of Bp, the indicated strains were incubated in the presence of a complement source and assessed for survival at different times post-incubation. A serum-sensitive *E. coli* was completely viable in the absence of complement or in heat-inactivated sera, but was killed within 2 h of incubation in 20% and 40% serum, confirming the bactericidal activity of complement in the serum source (Figure 5). In contrast, Bt, Bp, and the acapsular mutant survived even at high concentrations of serum up to 6 h post-incubation. Thus, neither the varied levels of complement deposition observed on the different bacterial strains nor the presence of the polysaccharide capsule were responsible for the resistance to direct complement-mediated killing displayed by the *Burkholderia* strains. Alternatively, a Bp mutant that is unable to express LPS displayed 100- to 1000-fold greater serum sensitivity compared to wild type, suggesting that the polysaccharide components of LPS are important for resisting complement-mediated direct killing, which is in agreement with previous reports [62], [86].

### Serum opsonization of Bp and Bt results in increased bacterial uptake, but no effect on clearance by macrophages

**Figure 6:**
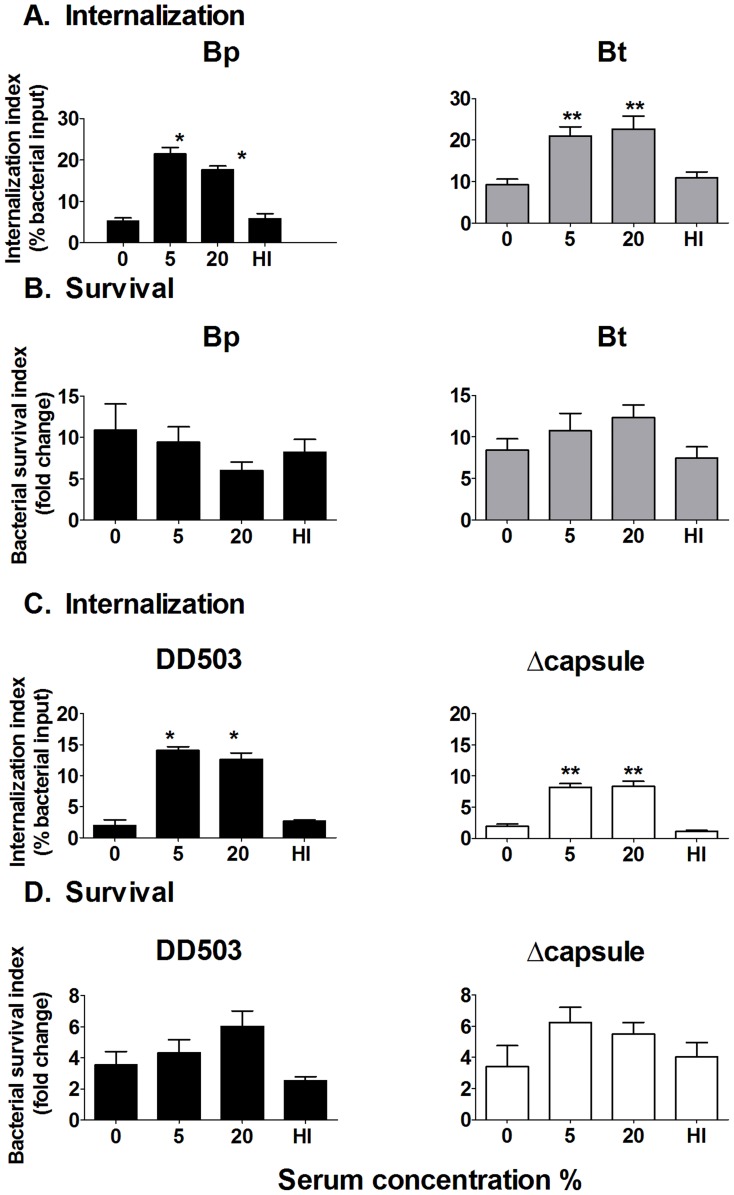
Effect of serum opsonization on uptake/persistence of *Burkholderia* strains in macrophages. Bacterial strains (2×10^5^) were opsonized with the indicated normal serum concentration before adding to macrophages (MOI = 1) for 1 h. After removing extracellular bacteria, macrophages were harvested at 3 and 10 h post-infection to determine bacterial uptake (**A** and **C**) and survival (**B** and **D**); bacterial numbers were determined by dilution plating. * p<0.05 to Bp 0%, ** p<0.05 compared to Bt 0% or acapsular mutant 0% as determined by one-way ANOVA; error bars represent standard error mean. (n≥6).

To determine the effect of differential levels of complement deposition between Bp and Bt on phagocytosis and survival within macrophages, intracellular growth assays were performed. Compared to unopsonized or heat-inactivated serum-opsonized bacteria, opsonization of Bp and Bt with 5% and 20% normal serum significantly enhanced bacterial uptake (≥2 fold increase) by macrophages (Figure 6A). When intracellular killing was assessed, both Bp and Bt displayed similar resistance to intracellular clearance irrespective of the level of complement opsonization (Figure 6B). To directly delineate whether capsule confers any specific effects on uptake/killing of complement-opsonized bacteria, similar serum opsonization studies were performed comparing Bp and the acapsular mutant. Bp and the acapsular mutant demonstrated similar increased uptake by macrophages in presence of 5% and 20% serum compared to the respective unopsonized bacteria (Figure 6C). Both strains also showed substantial intracellular survival in the presence/absence of serum opsonization, similar to that reported for Bp and Bt (Figure 6D). These findings indicate that regardless of the levels of complement deposition on Bp or Bt, this opsonin is unable to elicit intracellular killing of either strain by macrophages, even though it can enhance bacterial uptake.

### Serum opsonization of Bp and Bt results in increased bacterial uptake and killing by neutrophils

**Figure 7:**
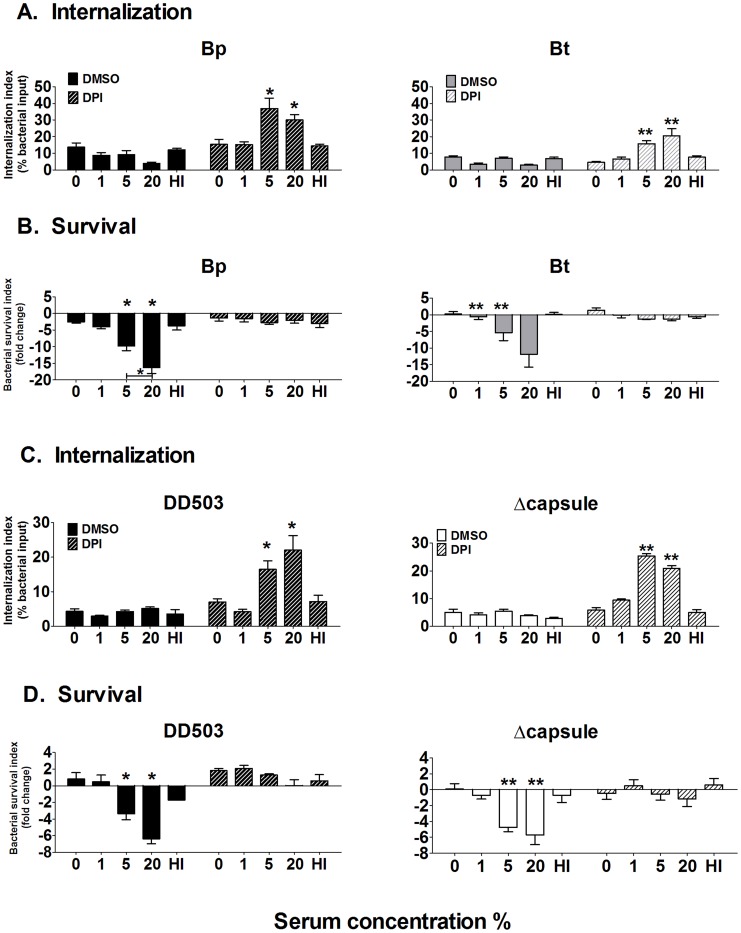
Effect of serum opsonization on uptake/persistence of *Burkholderia* strains in neutrophils. Neutrophils were treated with 10 µM DPI or DMSO (vehicle control) for 1 h prior to bacterial infection. Neutrophils were infected with the indicated serum-opsonized or unopsonized bacteria and the extracellular bacteria removed by washing 10 min post-infection. Infected neutrophils were harvested at (**A** and **C**) 10 min to measure bacterial uptake and at (**B** and **D**) 2 h to measure bacterial survival. Bacterial numbers were determined by dilution plating. DPI (diphenyleneiodonium); DMSO (Dimethyl sulfoxide; vehicle control); HI- Heat-inactivated. * compared to Bp 0% DMSO and ** compared to Bp acapsular mutant 0% DMSO as determined by one-way ANOVA; error bars represent standard error mean. (n≥6).

To determine if complement opsonization can affect uptake and killing by neutrophils, Bp and Bt were pre-incubated with normal serum and assessed in the presence/absence of the ROS-inhibitor DPI. Without blocking ROS (DMSO vehicle control), complement opsonization had no effect on bacterial uptake (Figure 7A). However, opsonization with 5% and 20% normal serum provided a significant increase in uptake when ROS was blocked, indicating that complement deposition both enhanced bacterial uptake and elicited substantial ROS production that was capable of reducing intracellular bacterial numbers within 10 min of co-incubation (Figure 7A). The lack of enhanced uptake of unopsonized, 1%-opsonized and heat-inactivated serum-opsonized bacteria confirmed the involvement of complement in increasing internalization. By 2 h post-infection, neutrophils infected with 5%- and 20%-opsonized Bp and Bt showed significantly lower bacterial numbers compared to bacteria opsonized with ≤1% serum (Figure 7B solid bars), suggesting that the complement levels deposited on Bp and Bt in the presence of ≥5% serum can activate neutrophils to promote intracellular killing. DPI-treated neutrophils showed no differences in intracellular bacterial numbers irrespective of the amount of opsonization, suggesting that ROS-mediated killing is important for clearing these bacteria by neutrophils (Figure 7B; hatched bars).

To more directly assess the role of capsule in serum-mediated activation of neutrophils, similar experiments were performed comparing Bp with an acapsular mutant. Again, DMSO-treated neutrophils displayed similar low uptake of Bp and the acapsular mutant irrespective of serum opsonization (Figure 7C; Solid bars), whereas ROS-blocked neutrophils could efficiently phagocytose both Bp and the acapsular mutant when opsonized with ≥5% serum (Figure 7C; hatched bars). No enhanced uptake was observed on infection with unopsonized, 1%-opsonized and heat-inactivated serum-opsonized bacteria, indicating that bacteria opsonized with ≥5% serum elicit enhanced uptake by neutrophils, regardless of capsule production. Assessment of intracellular clearance in DMSO-treated neutrophils showed significant killing of both strains when opsonized with ≥5% normal serum, where DPI-treated neutrophils displayed no ability to kill even opsonized bacteria (Figure 7D). Altogether, these studies indicate that neutrophils can efficiently kill even encapsulated Bp if these bacteria possess a minimum threshold of complement deposition on their surface (i.e. ≥5% normal serum), and that capsule does not directly affect these killing mechanisms.

### Sufficient complement opsonization of Bp and Bt leads to rapid ROS production by neutrophils

**Figure 8:**
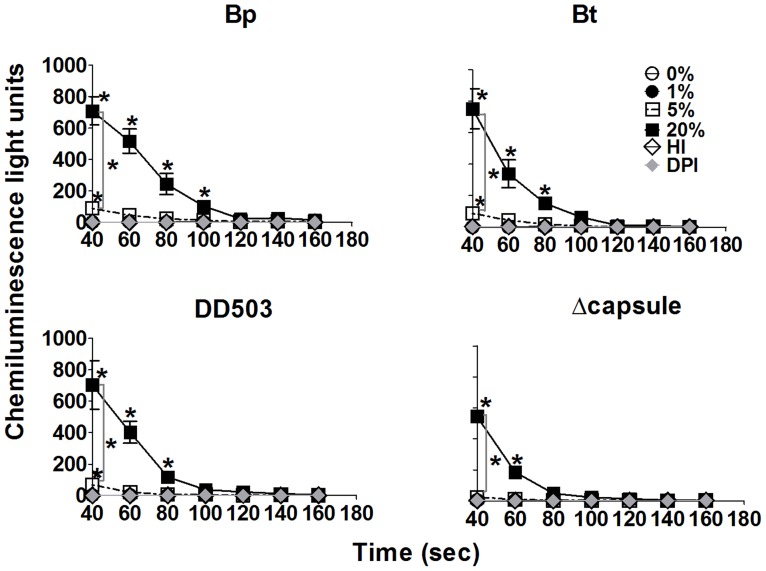
Reactive oxygen species (ROS) production by neutrophils. Neutrophils were cultured in complete RPMI at 2×10^6^ cells per well in a 6-well plate for 2 h. Adherent neutrophils were recovered and re-seeded at 2×10^5^ cells per well in a 96-well plate in PBS containing Ca^2+^ and Mg^2+^ in the presence or absence of DPI (10 µM). Neutrophils were pretreated with luminol (200 µM) for 20 min before infecting with bacteria (MOI = 5) pre-incubated with the indicated non-immune serum concentrations. Luminescence was assessed beginning at 40 s post-infection, with detection every 20 s for 10 min. * p<0.05 compared to respective 0% as determined by one-way ANOVA; error bars represent standard error mean. (n = 4).

Because major differences in bacterial uptake were seen within 10 min only for neutrophils treated with DPI, experiments were performed to confirm if complement-opsonization elicited a rapid ROS response that was responsible for this killing using a luminol-based chemiluminescence assay. Co-culture of neutrophils with Bp or Bt opsonized with ≤1% or heat-inactivated serum did not elicit ROS production (Figure 8; upper panels). However, bacteria pre-incubated with ≥5% normal serum elicited very rapid ROS production, and responses to 20% opsonized bacteria were significantly greater than those to 5% serum (Figure 8; upper panels). The rapidity was notable in that the highest ROS levels were observed at 40 s post-exposure, which was the earliest time point that luminescence readings could be obtained after adding the bacteria. No differences in the magnitude or kinetics of ROS production were observed between Bp and Bt at any serum condition tested. In both cases, DPI was completely able to block ROS production by neutrophils, even in response to 20% serum-opsonized bacteria. To more directly assess the role of capsule in ROS production, similar experiments were performed comparing neutrophil activation in response to Bp and the acapsular mutant. Similar ROS levels were produced by neutrophils infected with 20% opsonized Bp and the acapsular mutant (Figure 8; lower panels). However, while Bp opsonized with 5% serum did elicit significant ROS production, the acapsular mutant opsonized with 5% serum was unable to induce similar ROS production by neutrophils. It is possible that the acapsular mutant elicited a quicker ROS response that was largely missed due to the inability to measure luminescence concurrent with the addition of bacteria. Together, these findings indicate that Bp and Bt both elicit a similar, rapid activation of ROS that is independent of capsule production, but required a minimal threshold of complement deposition that was similar to that required for inducing efficient killing of these bacteria (Figure 7).

### Antibody binding and serum opsonization have no additive effect on bacterial killing by macrophages

**Figure 9:**
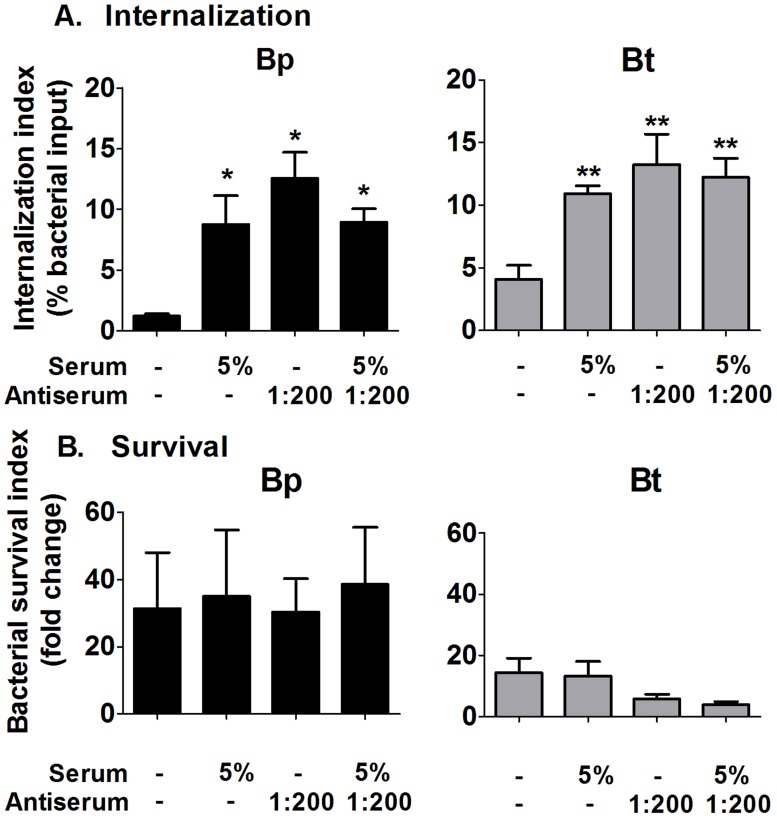
Effect of antibody and complement opsonization on bacterial persistence in macrophages. 2×10^5^ macrophages were infected with serum (5%) and/or antibody (1∶200) opsonized Bp or Bt (MOI = 1) for 1 h before removing the extracellular bacteria. Macrophages were harvested at 3 h and 10 h post-infection to determine bacterial uptake (**A**) and survival (**B**), and bacterial numbers determined by dilution plating. * p<0.05 compared to Bp 0%, ** p<0.05 compared to Bt 0% as determined by one-way ANOVA; error bars represent standard error mean. (n = 4).

To address whether antibody binding could enhance complement-mediated effects on macrophages, intracellular uptake and killing assays were performed with the indicated combinations of antisera and complement sources. Macrophages co-incubated with Bp or Bt opsonized with either specific antibodies or complement alone displayed significantly increased uptake compared to unopsonized bacteria (Figure 9; upper panels). However, the combination of antisera and complement showed no enhancement in uptake compared to either opsonin alone. For intracellular clearance, neither the addition of antisera or complement alone, nor the combination of the two opsonins, was able to mediate clearance of Bp or Bt (Figure 9; lower panels). Thus, there is no additive effect of specific antibodies and complement opsonization on clearance of Bp or Bt by macrophages *in vitro*.

### Antibody binding and serum opsonization have no additive effect on bacterial killing by neutrophils

**Figure 10:**
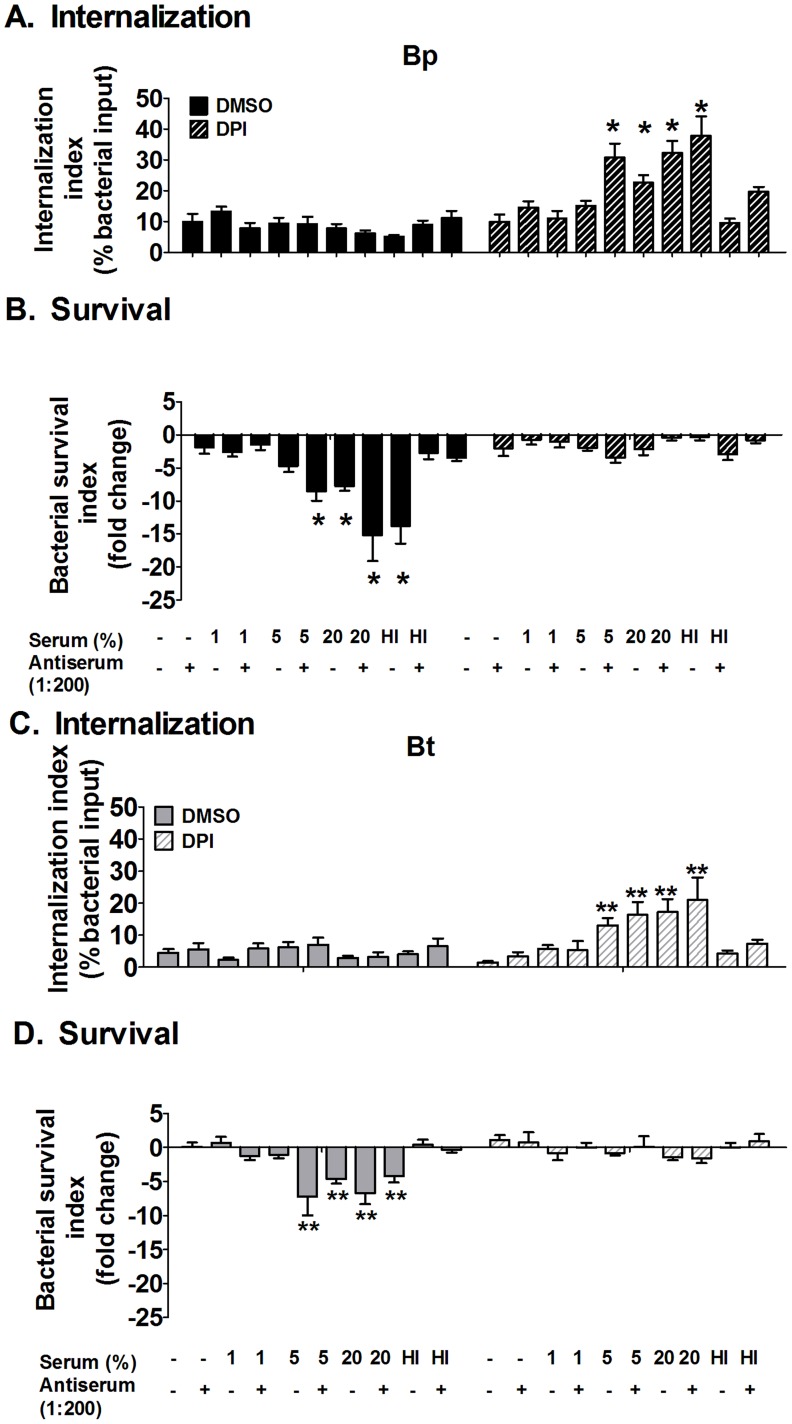
Effect of antibody and complement opsonization on bacterial persistence in neutrophils. Neutrophils were treated with 10 µM DPI or DMSO (vehicle control) for 1 h before co-incubation with antiserum (1∶200) and/or serum (1–20%)-opsonized Bp (**A–B**) or Bt (**C–D**) for 10 min before removing extracellular bacteria. Neutrophils were harvested at 10 min (**A** and **C**) post-infection to measure bacterial uptake and at 2 h (**B** and **D**) to measure bacterial survival. Bacterial numbers were determined by dilution plating. HI- Heat-inactivated. * compared to Bp 0% DMSO as determined by one-way ANOVA; error bars represent standard error mean (n = 3).

Similar experiments were performed to detect whether antibody binding could augment complement-mediated opsonic effects for neutrophils. Unlike macrophages, when ROS was blocked there was no significant difference in neutrophil uptake of unopsonized- and antiserum-opsonized Bp (Figure 10A; hatched bars). Addition of ≥5% normal serum increased neutrophil uptake of Bp, however the addition of antisera provided no additional enhancement at any concentration of normal serum. The addition of antisera alone did not increase intracellular killing of Bp, and when added with complement displayed no additional killing over that of complement alone (Figure 10B; solid bars). Similar assessments of Bt showed the same effects of antisera as that displayed by Bp, suggesting that capsule has no influence on the internalization and killing effects in response to antibody binding (Figure 10C–D). Overall, the findings from this section suggest that antibody opsonization does not augment the internalization or killing of Bp by neutrophils over that observed by complement alone.

### Bacterial opsonization with specific antibodies and serum does not have any additive effect on ROS production by murine neutrophils

**Figure 11:**
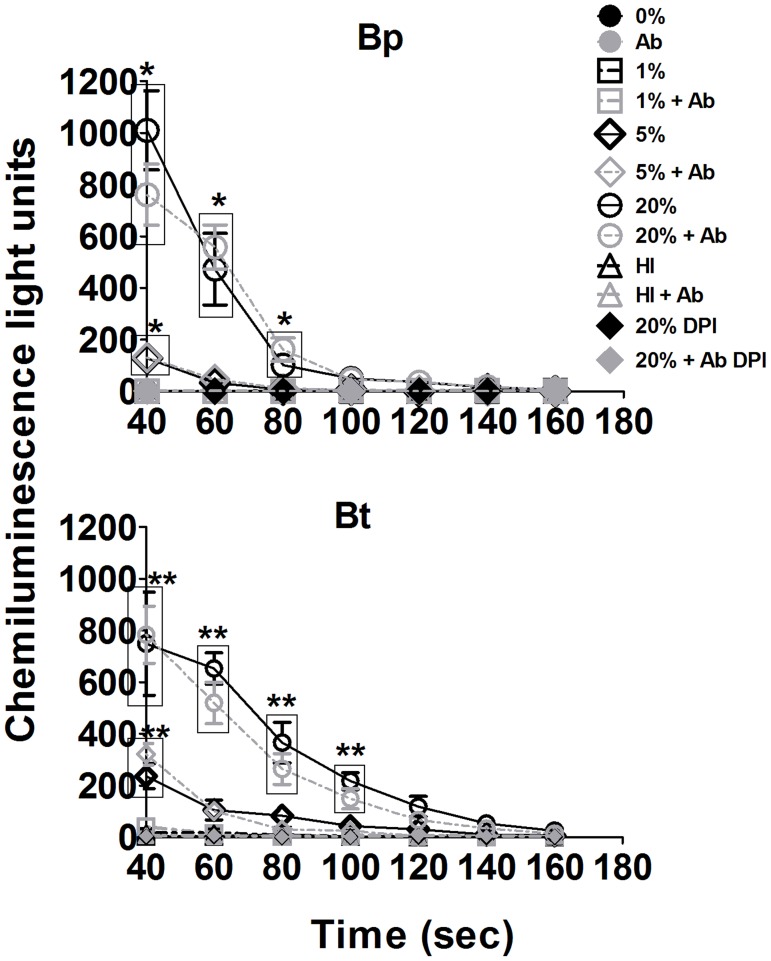
Effect of antibody and complement opsonization on ROS production by neutrophils. Neutrophils were cultured in complete RPMI at 2×10^6^ cells per well in a 6-well plate for 2 h. Adherent neutrophils reseeded at 2×10^5^ cells per well in a 96-well plate and assessed for luminescence after treatment with Bp or Bt opsonized with the indicated concentrations of normal serum and/or antisera (1∶200), as described in Figure 8. * p<0.05 compared to Bp 0%, ** p<0.05 compared to Bt 0% as determined by one-way ANOVA; error bars represent standard error mean. (n = 3).

Luminescence-based assays were performed to determine whether antibodies had any direct or synergistic effect on ROS responses to Bp and Bt by neutrophils. Antibody opsonization alone did not induce ROS production by neutrophils in response to Bp or Bt (Figure 11). Addition of antisera to Bp or Bt in the presence of a complement source was unable to enhance ROS production over that of bacteria opsonized with normal serum alone. Thus, there was no additive effect of complement and antibody on ROS production by neutrophils.

### Macrophages require IFNγ pre-activation to clear Bp and Bt independent of serum opsonins

**Figure 12:**
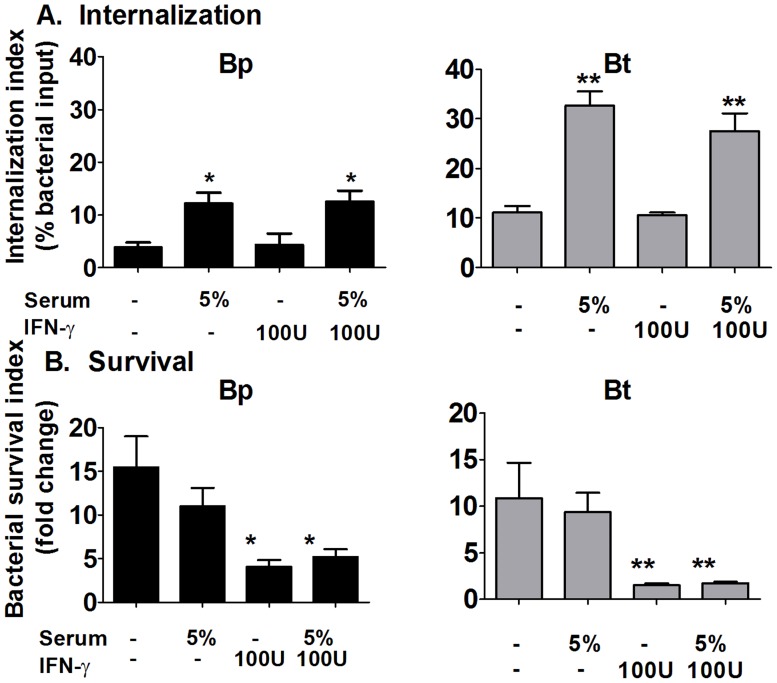
Effect of IFNγ pre-activation on bacterial clearance by macrophages. 2×10^5^ Bp or Bt were opsonized with serum (5%) before infecting IFNγ (100 U) -pretreated macrophages (MOI = 1) for 1 h. After removing extracellular bacteria, macrophages were harvested at 3 and 10 h post-infection to determine bacterial internalization (**A**) and survival (**B**) by dilution plating. * p<0.05 compared to Bp untreated, ** compared to Bt untreated as determined by one-way ANOVA; error bars represent standard error mean. (n≥6).

Previous studies suggest that T cell help and/or IFNγ is critical for controlling melioidosis [87]. Because IFNγ is a good stimulant of macrophages *in vitro* as well as *in vivo*, we assessed the effects of IFNγ on macrophage clearance of opsonized versus unopsonized Bp and Bt. Serum opsonization alone increased the uptake of Bp and Bt, but did not increase clearance of either species (Figure 12A–B). Pretreatment with IFNγ did not enhance uptake of either bacteria, but IFNγ-pretreated macrophages were successful in reducing the number of intracellular Bp and Bt (Figure 12B), and this effect was independent of serum opsonization. Thus, pre-activation with IFNγ allows macrophages to efficiently clear Bp and Bt, but these effects are independent of any activity provided by complement opsonization.

## Discussion

*Burkholderia pseudomallei* (Bp) infections can be highly lethal due to its infectivity through multiple routes, existence of various predisposing conditions, variability of symptoms displayed, and lack of effective therapies. Categorization of Bp as a potential bioterrorism agent and the spread of its endemic locations have increased its importance as a global threat. Thus, there is a great need to develop better preventive and curative strategies for Bp infections. Because Bp can persist within multiple host cell types, it is important to better understand the immune response elicited during Bp infection and identify mechanisms to enhance their protective properties. The innate immune responses are critical for containing Bp infections, particularly as acute disease can prove lethal within a few days of infection and before the development of the adaptive responses. In these studies, we sought to delineate the bactericidal capacities of two important immune cell types, macrophage and neutrophils, and whether opsonization of Bp with complement or antibodies can enhance these protective abilities. Additionally, the direct effects of the bacterial capsule on evasion of these immune mechanisms were assessed for both cell types.

In the absence of any serum opsonins, Bp, Bt, and a Bp acapsular mutant were phagocytosed by both macrophages and neutrophils at similar rates, and were subsequently able to efficiently persist within both cell types at similar levels. This indicated that the capsule produced by Bp is not anti-phagocytic and is not essential for evasion of intracellular killing mechanisms elicited by either phagocyte under these conditions, which is different from previous speculation [63]. These findings are also contrary to prior reports indicating that Bt is cleared more efficiently by macrophages compared to Bp [60], [61]. To test the discrepancies between these studies, we performed experiments under conditions similar to the published data, and demonstrated that the differences between Bp and Bt could be explained by the presence of low antibiotic concentrations that were maintained during macrophage co-culture experiments. It has been shown with *Listeria monocytogenes* that the presence of even low levels of antibiotics in macrophage medium leads to increased antibiotic concentration within these cells due to their pinocytic properties, allowing an artificial intracellular killing effect [80], [83]. Using our assay system, where antibiotics are excluded during the majority of the intracellular incubation, both Bp and Bt were quite resistant to intracellular clearance by either macrophages or neutrophils in the absence of any serum opsonins. This suggests that even the relatively avirulent Bt possesses the rudimentary virulence machinery to persist within these immune cells independent of further external immune activation.

Upon entry into host tissues, bacteria are exposed to soluble immune mediators that can bind to the bacterial surface and subsequently “opsonize” them, such that they are more readily recognized by receptors on macrophage and neutrophils. This binding usually results in enhanced phagocytosis of the bacteria and/or activation of the phagocytes to enhance their various killing mechanisms to affect intracellular clearance. This study assessed two of the most potent serum opsonins, complement and antibodies, as to their abilities to promote phagocyte-mediated clearance. Bp and Bt exhibited similar, high levels of antibody-binding when exposed to their respective antisera, whereas Bt displayed significantly higher levels of complement C3-complexes on its surface compared to Bp, particularly in the presence of ≥5% non-immune serum. Exposure to antibodies alone had no effect on the abilities of neutrophils to phagocytose or kill Bp and Bt. However, neutrophils exposed to complement-opsonized bacteria showed significantly enhanced uptake and intracellular clearance of Bp and Bt, but only if opsonized with ≥5% normal serum. Alternatively, neutrophils exposed to Bp and Bt opsonized with antibodies alone displayed no increase in phagocytosis and killing, and even though antibody binding could enhance complement deposition on bacterial surfaces, this provided no additional enhancement compared to the effects provided by complement alone. All opsonizing conditions that elicited intracellular clearance by neutrophils were also able to produce a potent and rapid ROS response compared to non-productive opsonization conditions. This infers that antibody-opsonization alone is insufficient to elicit ROS from neutrophils, whereas sufficient complement-opsonization efficiently elicits ROS, leading to killing of intracellular *Burkholderia* sp. This difference does not appear to be due to the absolute number of bacteria ingested, since antibody- and/or complement-opsonization of the same number of bacteria elicits similar internalization of *Burkholderia* sp. (Figure 10A) and ROS production, if generated, within 2 min of inoculation (Figure 11). Previous studies have reported mixed results as to the ability of neutrophils to kill Bp. However, a more recent study indicated that human neutrophils could efficiently clear Bp, but only if they displayed at least a minimal threshold of complement deposition on its surface [62]. Our findings are most similar to this study, although the kinetics of murine ROS appears to be faster, of shorter duration, and of lower magnitude compared to that exhibited by human neutrophils, based on the *in vitro* findings. This is also in line with reports that depletion of neutrophils corresponds with more rapid mortality in Bp-infected mice [48], and is similar to the increased mortality seen in infection of ROS-deficient mice [39]. Overall, our findings solidify that neutrophils appear to be quite important for effective control of Bp infections, but only if mechanisms exist so that sufficient complement deposition can be directed onto the bacterial surface that allows rapid elicitation of ROS.

Since macrophages and neutrophils possess a similar spectrum of complement receptors and Fc receptors associated with mediating opsonin-enhancing immune properties, one can speculate that macrophages should show a similar enhancement of antibacterial mechanisms to opsonized Bp as neutrophils [88]–[90]. However, our studies indicate that macrophages are much less affected by these serum opsonins bound to *Burkholderia* spp. compared to neutrophils. Similar to neutrophils, complement opsonization conferred by ≥5% normal serum led to increased uptake of both Bp and Bt by macrophages. However, all tested serum concentrations were unable to confer enhancement of intracellular clearance by macrophages, even though the bacterial surface was relatively saturated with complement complexes that were able to elicit neutrophil-mediated killing. Antibody-binding alone was able to increase bacterial uptake, but could not elicit killing of either bacterial strains, and though antibody binding could substantially enhance complement deposition, these heightened levels were still unable to confer macrophage killing of Bp or Bt. Although none of the serum opsonins could enhance bacterial killing, pre-incubation of macrophages with IFNγ did elicit intracellular clearance that was independent of opsonization with either complement or antibody, confirming the general immune competence of our macrophage population. The reasons why serum opsonins are unable to confer macrophage killing of Bp, as opposed to neutrophils, is currently not well-understood. Macrophages are generally not major producers of ROS compared to neutrophils [91], [92], particularly in the absence of INFγ, and our own assessment detected very little ROS production by Bp-exposed macrophages irrespective of opsonization (data not shown). RNI production is often associated with macrophage-mediated killing of intracellular bacteria and Bp is sensitive to RNI-associated compounds [93]. However, Bp is reported to suppress RNI [94], and our studies indicate that serum opsonization does not enhance nitric oxide activity (data not shown). Bp has been reported to escape the phagosome as early as 15 min post-infection and subsequently escape into the cytoplasm, where it is protected from most phagolysosomal-associated killing mechanisms [23], [24]. It is likely that the rapidity of ROS production by neutrophils in response to complement-opsonized Bp allows these bacteria to be killed before they can escape into the cytoplasm. Alternatively, it appears that macrophages require different immune mediators that can activate bactericidal mechanisms capable of killing Bp after escaping into the cytoplasm, such as IFNγ [87], [95] or NOD-protein [96], [97] and NOD-like receptor-associated activation [98] that can promote inflammasome-mediated pyroptosis [99], [100]. Thus, even the presence of substantial *Burkholderia*-specific antibodies and other serum opsonins appears unable to promote Bp or Bt clearance by macrophages in the absence of IFNγ.

Multiple studies have shown that at least one of the polysaccharide capsules (i.e. capsular polysaccharide I; CPS I) produced by Bp is essential for allowing infection in susceptible hosts [64], but the specific virulence properties attributed to this capsule has been varied. Our *in vitro* studies have assessed many of these putative properties and delineated which appear responsible for evasion of direct and phagocyte-mediated killing mechanisms. While the Bp capsule has been speculated to be anti-phagocytic, our findings indicate that acapsular and capsular Bp are taken up similarly by both neutrophils and macrophages, and the presence of complement deposition increases uptake irrespective of possessing a capsule. The capsule also does not protect from bactericidal mediators produced by neutrophils and macrophages, since capsular and acapsular strains displayed similar susceptibilities to phagocyte killing. Capsule does not confer protection from direct complement-mediated killing, as both Bp and the acapsular bacteria possess similar resistance to killing, even though the levels of complement deposition was significantly higher in the acapsular strains. However, a significant level of serum sensitivity was displayed by the ΔLPS mutant, indicating that LPS does provide protection from the direct killing mechanisms elicited by complement deposition [62]. In certain bacteria it is suggested their LPS binds complement components associated with the membrane attack complex at a distance from the bacterial surface, thus not allowing insertion into the bacterial outer membrane [101], and such a mechanism could explain why both Bp and Bt are serum-resistant, as they produce similar type II O-antigenic polysaccharide [64], [86], [102]. It is possible that the most prominent effect mediated by capsule was the ability to reduce complement deposition on the outer surface, which has also been reported by others [62], [63]. The significance of this property is reflected in our finding that complement deposition is required for neutrophil-mediated killing of Bp, but only if a certain level of deposition is achieved (i.e. the activities that occur in response to ≥5% non-immune serum, which also elicits the ROS response associated with killing). A number of pathogenic microbes possess polysaccharide capsules that are associated with inhibiting complement deposition [103]–[106]. In many cases, the capsule itself is not resistant to complement deposition, but often is capable of binding host soluble complement regulatory proteins (e.g. Factor H, C4 binding protein, C1 inhibitor, etc.) via surface-exposed sialic acid or glycosaminoglycans that can bind these proteins in much the same way that host cells utilize them to prevent deposition on their own tissues [105], [106]. These bacteria can then utilize the proteins regulatory properties to promote degradation of C3 complexes into components that lack the ability to enzymatically amplify further complement activation, thus vastly limiting further complement activation to levels required for optimal opsonization. Also, certain capsules lack phase-variable phosphorocholine residues that can be bound by host C-reactive protein, which can subsequently bind complement component C1q to activate complement, and thus evade this activation pathway [107]. Several encapsulated and acapsular bacteria produce disparate outer membrane proteins that can directly attach to host complement regulatory proteins and subsequently use them to prevent complement amplification [108]–[110]. There have been no reports of Bp utilizing host complement regulatory proteins to resist complement deposition. However, our findings and those of others are suggestive that such capsular interactions with these regulators could best explain the substantial protective capabilities of Bp capsule *in vivo*. Identification of such pathways might provide targets for therapies to prevent melioidosis [111].

This study represents the first side-by-side comparison of macrophages and neutrophil responses to Bp, and delineates the immune mechanisms that are required to allow these early immune cell responders to efficiently recognize and kill Bp. The innate activation of the complement cascade appears to have the potential to opsonize Bp sufficiently to eradicate these pathogens, but only if enough deposition occurs to elicit ROS activity by neutrophils. It is possible that this minimal activation level is not normally achieved *in vivo*, either due to direct suppressive properties of the capsule of its ability to harness host complement regulatory proteins. While antibodies did not display synergistic or additive activation *in vitro*, it is possible that the observed antibody-mediated enhancement in complement deposition is necessary to achieve the minimum threshold stimulatory levels on Bp *in vivo*, whereas Bt attains these activating levels even in the absence of specific antibodies and are thus avirulent even in immunologically naïve hosts. This is supported by reports that passive administration of capsule-specific monoclonal antibodies can provide some protection from even aerosol challenge with Bp, but does not provide sterilizing immunity [112]–[114]. While this antibody and complement-mediated opsonization appears sufficient for directing neutrophil-mediated killing of extracellular Bp, bacteria that are engulfed by macrophages present a different problem. Even with serum opsonization, macrophage-residing Bp are able to replicate, persist, and potentially migrate throughout the host in these long-lived cells until they spread to adjacent cells via actin-based motility and giant cell formation, potentially without exposure to the external environment that contains these soluble opsonins [24]. Overall, our findings suggest that an ideal melioidosis therapy should be able to generate antibodies capable of enhancing complement deposition to mediate neutrophil-mediated clearance, as well as eliciting immune cell populations capable of secreting sufficient IFNγ (i.e. NK cells and certain T lymphocyte populations), and thus provide macrophages adequate activation to clear intracellular Bp, as well as those residing within other cell types. Such complete immune activation may be critical for both controlling acute infections, as well as eradicating persistent populations that are responsible for chronic and recrudescent melioidosis.
